# Radiomics nomogram for prediction of microvascular invasion in hepatocellular carcinoma based on MR imaging with Gd-EOB-DTPA

**DOI:** 10.3389/fonc.2022.1034519

**Published:** 2022-11-01

**Authors:** Shuai Zhang, Chongfeng Duan, Xiaoming Zhou, Fang Liu, Xin Wang, Qiulin Shao, Yuanxiang Gao, Feng Duan, Ruirui Zhao, Gang Wang

**Affiliations:** ^1^ Department of Radiology, The Affiliated Hospital of Qingdao University, Qingdao, China; ^2^ Operating Room, The Affiliated Hospital of Qingdao University, Qingdao, China

**Keywords:** hepatocellular carcinoma, microvascular invasion, radiomics, gadoxetic acid-enhanced mri, nomogram

## Abstract

**Objective:**

To develop a radiomics nomogram for predicting microvascular invasion (MVI) before surgery in hepatocellular carcinoma (HCC) patients.

**Materials and Methods:**

The data from a total of 189 HCC patients (training cohort: n = 141; validation cohort: n = 48) were collected, involving the clinical data and imaging characteristics. Radiomics features of all patients were extracted from hepatobiliary phase (HBP) in 15 min. Least absolute shrinkage selection operator (LASSO) regression and logistic regression were utilized to reduce data dimensions, feature selection, and to construct a radiomics signature. Clinicoradiological factors were identified according to the univariate and multivariate analyses, which were incorporated into the final predicted nomogram. A nomogram was developed to predict MVI of HCC by combining radiomics signatures and clinicoradiological factors. Radiomics nomograms were evaluated for their discrimination capability, calibration, and clinical usefulness.

**Results:**

In the clinicoradiological factors, gender, alpha-fetoprotein (AFP) level, tumor shape and halo sign served as the independent risk factors of MVI, with which the area under the curve (AUC) is 0.802. Radiomics signatures covering 14 features at HBP 15 min can effectively predict MVI in HCC, to construct radiomics signature model, with the AUC of 0.732. In the final nomogram model the clinicoradiological factors and radiomics signatures were integrated, outperforming the clinicoradiological model (AUC 0.884 vs. 0.802; p <0.001) and radiomics signatures model (AUC 0.884 vs. 0.732; p < 0.001) according to Delong test results. A robust calibration and discrimination were demonstrated in the nomogram model. The results of decision curve analysis (DCA) showed more significantly clinical efficiency of the nomogram model in comparison to the clinicoradiological model and the radiomic signature model.

**Conclusions:**

Depending on the clinicoradiological factors and radiological features on HBP 15 min images, nomograms can effectively predict MVI status in HCC patients.

## Introduction

Hepatocellular carcinoma (HCC) is the most prevalent cancer in China, with a high fatality rate (1, 2). Despite the surgical resection adopted as an effective treatment for HCC, recurrences remain common (3), which are experienced by approximately 70% of liver resection patients within five years, and approximately 25% of liver transplant patients (4). The microvascular invasion (MVI) refers to the tumor invasion in small intrahepatic vessels, covering portal veins, hepatic vessels, and lymphatic vessels (5). MVI in HCC is considered a feature of histologically generated case changes, implying the early postoperative recurrence with correspondingly lower survival (6). It is critical to accurately identify MVI in patients with HCC for developing appropriate treatment options. Surgery with wide margins is considered the best option for patients at high risk for MVI (7). However, in contrast to macrovascular invasion that can be detected by diagnostic imaging, MVI can only be diagnosed by pathologic evaluation currently. Thus, a quantitative method urgently required for preoperative prediction of MVI.

It has been demonstrated that by converting medical images into higher quality, quantifiable and mineable data, the radiomic features can serve as the diagnostic and prognostic markers for cancer phenotypes and tumor microenvironments (8, 9). Our previous study (10) has indicated that MVI could be predicted by radioactivity in the hepatobiliary phase (HBP) on Gd-EOB-DTPA magnetic resonance imaging. However, further integration with clinical data and radiological features is required for physicians to accept its full and robust role in patient management. As a direct extension of our previous work, the objective of this study is to predict the state of MVI in HCC patients by creating a nomogram that incorporated the clinicoradiological factors and radiomics signatures.

## Materials and methods

### Patients

This retrospective study was approved by an institutional review board, with the patient’s own informed consent waived. 189 consecutive HCC patients from the period January 2015 to April 2022 were enrolled. The cohort was divided into a training set from January 2015 to May 2020, with 82 MVI+ patients (76 men and 6 women; range, 37-79 years) and 59 MVI- patients (40 men and 19 women; range, 35-77 years) and a validation set from May 2020 to April 2022, with 29 MVI+ patients (25 men and 4 women; range, 38-76 years) and 19 MVI- patients (14 men and 5 women; range, 39-75 years). The inclusion criteria were: (1) Gd-EOB-DTPA-enhanced MRI performed within one month before surgical resection; (2) The postoperative pathological features met the clinical criteria for HCC. The criteria for exclusion were: (1) patients receiving liver cancer-related treatment before surgery; (2) patients with macrovascular invasion on MRI; and (3) insufficient images for radiomic analysis.

### MR Techniques

MR imaging was performed on all participants with a 3.0 T scanner (GEHCGEHC, GE medical systems, Waukesha, WI). All patients received the GdEOB-DTPA (Primovist, Bayer HealthCare, Berlin, Germany) with 0.1 mL/kg (0.025 mmol/kg). After 5 minutes, 10 minutes, and 15 minutes (i.e., HBP are the three different time periods mentioned above, respectively) injected with the comparator agent, data on the inhibition of liver production by 3D fat-suppressed Liver Acquisition with Volumetric Acceleration (LAVA, GE Healthcare) sequence in the axial plane were collected. Contrasts for LAVA sequences include TR/TE, 2.5/1.1; slice spacing, 2.5 mm; thickness, 5 mm; reverse time, 5.0 ms; field of view, 380-450mm; Get the number of characters, 0.70; and the bandwidth, 976.6 kHz.

### Clinicoradiological risk factors

The clinical characteristics of the patients were recorded by our hospital’s HIS system, including patient age, gender, alpha-fetoprotein (AFP) level, presence of liver cirrhosis, and hepatitis B and C surface antigen (HBsAg) status (positive or negative). The MVI statue was obtained from the pathology report. The imaging, including diameter, halo sign, shape, border, radiocapsule, necrosis, and tumor/liver signal ratio, was carried out based on MRI findings by two radiologists independently through the collection of pictures and communication systems (PACS).

Tumor diameter was defined as the largest diameter imaged by transverse at HBP 15 minutes; Halo sign was defined as a hypointense ring in the center of the lesion on HBP images; Tumor shape was classified as round or non-round, with the ratio of long diameter to short diameter less than 1.2 means round, otherwise, it means not round; Radiological capsule appearance was defined as hyperenhancing structures surrounding the tumor in the portal vein or at extension; tumor/liver signal ratio was expressed as the signal of the tumor/surrounding liver parenchyma on HBP images; necrosis was defined as high T2 and no enhancement in the tumor. To identify the single factor for MVI discrimination, univariate analysis was performed, and significant univariate factors (P<0.1) were entered into a multivariate logistic regression mode in the training cohort. P<0.05 was regarded as significant in the multivariate analysis.

### MR Radiomics analysis

**Figure 1:**
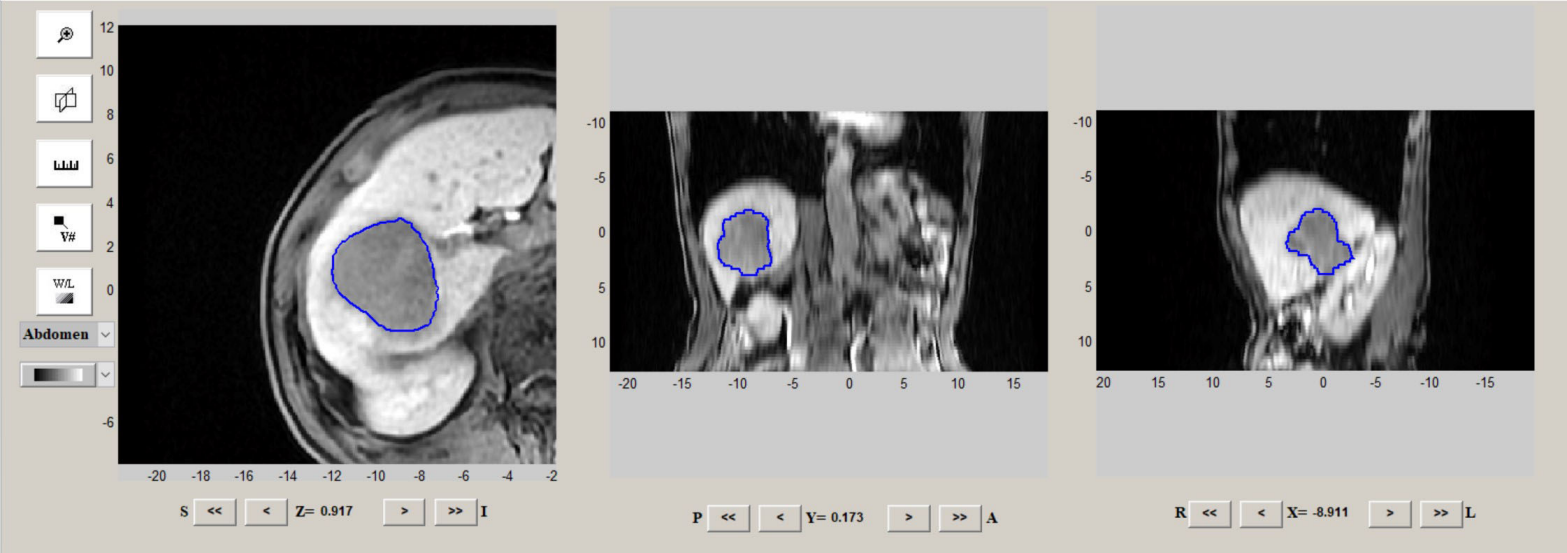
Example of ROIs delineation on HBP 15 min images by IBEX software.

Radiomics analysis mainly refers to tumor segmentation, feature extraction, feature selection, and model building and evaluation. The regions of interest (ROIs) were delineated on HBP 15 min images by IBEX software (http://bit.ly/IBEX). Tumor ROIs were manually segmented covering the whole tumor by two abdominal radiologists blinded to the pathology results (**Figure 1**). A total of 1768 MR image features of HBP 15 min from the tumor were analyzed using IBEX software. Radiomic parameters were determined depending on IBEX software, obtaining a total of 8 groups of parameters, each with different radiomics. To analyze and examine the reproducibility of the features extracted by repeated sequences, 30 tumor samples were randomly selected for the calculation of the intra-group correlation coefficient (ICC), with the features with ICC<0.80 excluded. The classification of images and the main filtering process were detailed in a previous study (10). The least shrinkage regression analysis and selection operator (LASSO) were adopted to select the most critical parameters obtained within 15 minutes of HBP. The combination of radiological features calculated by the LASSO coefficient weighting method was considered the radiomics score for each patient.

### Construction and evaluation of MVI prediction models

After univariate and multivariate logistic regression, the significant variables were selected to establish the clinicoradiological model. Radiomics signature model was built based on selected radiomics features. The nomogram model was constructed combining the clinicoradiological risk factors and radiomics signature. The potential predictive value of the three models was first assessed in the training cohort and then validated in the validation cohort by converted into quantifiable data using the area under the curve (AUC) in the receiver operating characteristic (ROC) curve, with the curves expanded for multiple contrasts by performing the Delong test on Bonferroni-adjusted p-values. AUC with 95% CI, precision, sensitivity and specificity was calculated.

### Construction and validation of the radiomics nomogram

The nomogram is calibrated by drawing a calibration curve in the training cohort. The Hosmer-Lemeshow test was carried out to analyze and determine the agreement between the MVI predicted by the nomogram and the actual MVI derived from the calibration curve. Decision curves were plotted to assess the clinical validity of nomogram in the combined training and validation cohorts.

### Statistical Analysis

SPSS (version 20, Chicago, IL, USA) and R (https://www.r-project.org/) were utilized as the statistical analysis tool. Only two-tailed in the case of p<0.05 was considered statistically significant.

## Results

### Construction of clinical radiological characteristics and clinical radiological models of patients

The clinicoradiological characteristics of the patients are detailed in **Table 1**. After univariate and multivariate analysis, it was determined that gender (odds ratio (OR) 6.06; 95% confidence interval (CI) 1.93–18.99), AFP level (OR 3.44; 95% CI 1.33–8.92), halo sign (OR 0.14; 95% CI 0.02–0.92), and shape (OR 0.12; 95% CI 0.05-0.31) can be chosen to construct clinical models.

**Table 1:** Comparisons of clinicoradiological characteristics in MVI (+) and MVI (-) patients.

Clinicoradiological characteristics	MVI (+) (N=82)	MVI (-) (N=59)	Univariate analysis		Multivariate analysis			
Odd ratios (95%CI)	p		Odd ratios 95%CI)		p
Clinical characteristics								
Age, (Median [range]), year	57[37-79]]	55[35-77]	1 (0.96-1.03)	0.841		–	–	
Gender (male/female)	76/6	40/19	6.02 (2.23-16.25)	<0.001		6.06 (1.93-18.99)	0.002	
Cirrhosis (present/absent)	81/1	55/4	5.89 (0.64-54.17)	0.117		–	–	
HBsAg (positive/negative)	75/7	54/5	0.99 (0.3-3.29)	0.99		–	–	
HCsAg (positive/ngative)	4/78	1/58	2.97 (0.32-27.3)	0.335		–	–	
AFP (> 400 ng/mL≤ 400 ng/mL)	32/50	10/49	2.98 (1.32-6.72)	0.009		3.44 (1.33-8.92)	0.011	
MR imaging features								
Diameter (Median [range]), milimetre	20.95[6-167]	23[3.36-106.7]	1.01 (1-1.02)	0.136		–	–	
Halo sign (present/absent)	2/80	6/53	0.22 (0.04-1.13)	0.071		0.14 (0.02-0.92)	0.04	
Shape (round/not round)	11/71	31/28	0.14 (0.06-0.32)	<0.001		0.12 (0.05-0.31)	<0.001	
Boundary (clear/unclear)	74/8	55/4	0.67 (0.19-2.35)	0.534		–	–	
Radiologic capsule (present/absent)	13/69	7/52	1.4 (0.52-3.75)	0.504		–	–	
Necrosis (present/absent)	28/54	17/42	1.28 (0.62-2.65)	0.503		–	–	
Tumor/liver signal ratio (mean ± SD)	0.5585±0.1659	0.52±0.1581	1.87 (0.23-15.06)	0.555		–	–	

P values were obtained from univariate and multivariate regression analyses between the MVI (+) and MVI (-) patients.

AFP alpha-fetoprotein, HBsAg hepatitis B surface antigen status, HCsAg hepatitis C surface antigen status, MVI, microvascular invasion.

### Radiomics signature calculation

A total of 1768 features were obtained from MR image features on HBP within 15 minutes. 356 radiomic features with most significant difference were then selected from the MVI+ and MVI- groups and introduced into a LASSO logistic regression model to screen out the most contributing features. Finally, 14 features with significant relation to MVI status were chosen for construction of the radiomics signature. Radiomics scores were calculated with the following formulas:

Radiomics score = -0.0144246× MedianAbsoluteDeviation-0.149397×5Percentile+0.00529663×Mass-6.210769×SphericalDisproportion-0.0005163601×4.7AutoCorrelation+0.02526177×1.7Contrast-0.008745637×9.4Contrast-1.448129×6.1DifferenceEntropy-0.1107169×4.7Dissimilarity-15.27011×8.4InverseDiffNorm+3.353539×1.1InverseVariance+4.034202×11.4InverseVariance-5.335912×12.4InverseVariance-0.607462×8.4MaxProbability.

### Performance of the models

**Figure 2:**
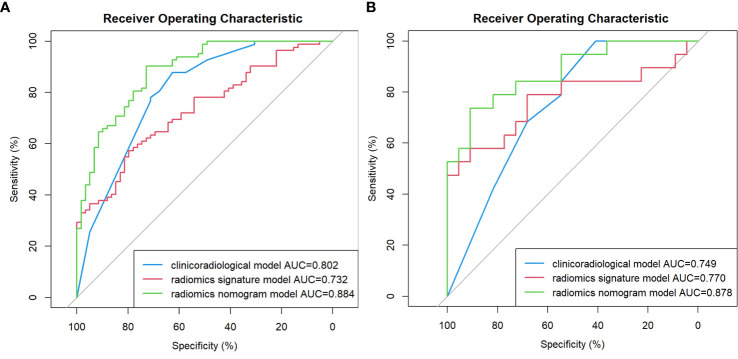
Comparison of receiver operating characteristic (ROC) curves for the prediction of microvascular invasion. ROC curves of the clinicoradiological model, the radiomics signature model and the radiomics nomogram model in the training and validation cohort **(A, B)**, respectively.

As shown in **Table 2** and **Figure 2**, In the training cohort, the AUC of the clinicoradiological model was 0.802 (95% CI: 0.730-0.875), radiomics signature model was 0.732 (95% CI: 0.650-0.813), and the nomogram model was 0.884 (95% CI: 0.790-0.924), with the Delong test results of the three models listed in **Table 2**. In the training cohort, the nomogram model was significantly better than the clinicoradiological model and radiomics signature model (P<0.001). In the validation cohort, the radiomics signature model and the nomogram model showed comparable discriminative power (AUC, 0.770 vs. 0.878, P = 0.0990), while the final nomogram model was significantly better than clinicoradiological model (AUC, 0.878 vs. 0.749, P = 0.0428).

**Table 2:** Predictive performance of the three models.

	Training cohort				Validation cohort			
	AUC (95%CI)	SEN	SPE	P	AUC (95%CI)	SEN	SPE	P
(1) Clinicoradiological model	0.802(0.730-0.875)	0.627	0.878		0.749(0.601-0.896)	0.409	1.000	
(2) Radiomics signature model	0.732(0.650-0.813)	0.797	0.573		0.770(0.613-0.928)	0.909	0.579	
(3) Radiomics nomogram model	0.884(0.790-0.924)	0.829	0.938		0.878(0.773-0.983)	0.909	0.573	
1 vs. 2				0.1868				0.8624
1 vs. 3				0.0002				0.0428
2 vs. 3				0.0003				0.0990

1 indicates clinicoradiological model; 2 indicates radiomics score model; 3 indicates radiomics nomogram model.

SEN sensitivity, SPE specificity, AUC area under the curve, CI confidence interval.

*P < 0.05 indicates a significant difference.

### Nomogram construction

**Figure 3:**
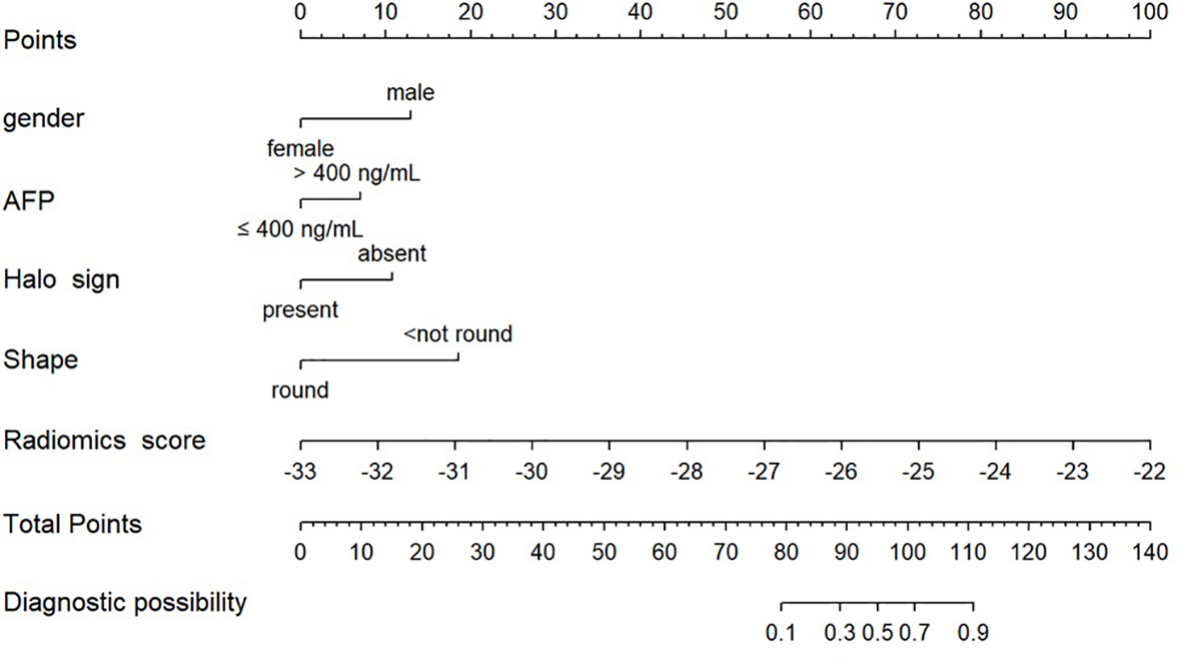
Radiomics nomogram combining the radiomics signature derived from HBP 15min MR images and clinicoradiological factors including gender, AFP, halo sign and shape for predicting MVI in the training cohort.

**Figure 4:**
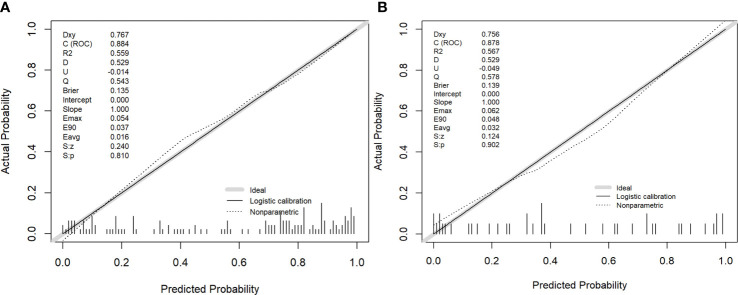
The performance of the nomogram was assessed by calibration curves in the training and validation cohort **(A, B)**, respectively. The y-axis represents the actual microvascular invasion (MVI) rate, the x-axis represents the predicted MVI possibility, and the diagonal dashed line indicates the ideal prediction by a perfect model.

**Figure 5:**
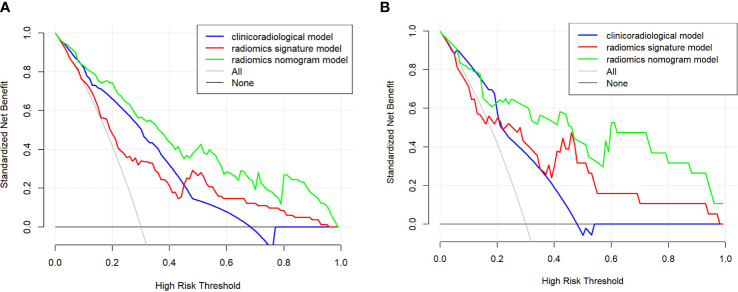
The clinical utility of the nomogram was evaluated by decision curves in the training and validation cohort **(A, B)**, respectively. In the decision curves, the black line indicates the net benefit of assuming that there are no patients with microvascular invasion (MVI), and the grey line indicates the net benefit of assuming all patients with MVI. The radiomics nomogram model (green line) provided a greater net benefit than the clinicoradiological model (blue line) and radiomics signature model (red line).

The nomogram model integrating clinicoradiological factors and radiomic signatures displayed robust predictive performance, so the calculated nomogram was adopted as the prediction graph (**Figure 3**). Acceptable calibrations of the nomogram are shown in **Figure 4**. The Hosmer-Lemeshow test suggested no significant difference between the predicted calibration curve and the MVI ideal curve in the training and validation cohorts (P = 0.450, P=0.761, respectively). In **Figure 5** the DCA results of the above three models in the training and validation cohorts are depicted. The nomogram model exhibited a larger net benefit in comparison to clinicoradiological model and radiomics signature model.

## Discussion

Previous studies indicated that MVI was the most robust independent predictor of recurrence and poor outcome for HCC (11, 12). Surgeons would be able to make better management decisions and improve prognostication if they were aware of MVI status before surgery. If the risk of predicted MVI indicates high, other alternative treatment options, such as the adjuvant therapy performed before surgery, should be considered or liver transplantation may not suitable for the patient (13). But for now, the predictive accuracy of MVI remains difficult, so we attempted to address this problem with radiomics.

Radiomics analysis is currently considered as a potential bridge connecting medical imaging and personalized medicine (14). The quantitative image processing can contribute to effectively evaluating the spatial relationship of pixel intensities (15) with a large role in medical practice and application value (16). As a relatively novel field, radiomics help to deeply mine medical imaging data by applying advanced computational methods, and the collected data can be further converted into quantitative data that can be applied to diagnosing the parameters of cancer, stage, prognosis, predicting treatment response, monitoring disease, etc. (8).

Some recent studies have demonstrated that the combined radiomics features can also play a predictive role in preoperative MVI in HCC patients (17–20). Consistently, we also found the good discrimination shown by radiomics features, as the AUC was 0.732. It is challenging to analyze and interpret the relationship between radiomic features and MVI status, considering more information maximized from radiographic analysis in comparison to visual inspection.

We found that gender can serve as an independent risk factor for MVI, which is obviously distinguished from previous studies (18, 21). The value of gender in predicting MVI in HCC has not been demonstrated, and further research is required. HCC is often associated with a higher level of AFP, which significantly increased in MVI patients. Our final study demonstrated AFP level as an independent risk factor for MVI, which is also consistent with previous conclusions (18, 22). Previous studies have also concluded that larger tumors significantly increased the risk probability of MVI in HCC (23, 24). However, this association was not indicated in our study, probably due to the selection bias. In addition, we found that the absence of halo signs and non-circular MR imaging features are the key predictor of MVI, which is consistent with previous studies (25–27). The results of observation on the pathological data indicated that among the current cases of MVI+ HCC, the most common is the single-nodular type and the multi-nodular type with additional nodular growth or fusion (28), which means the non-round tumor shape is the MR image feature of MVI+ HCC. In our previous study (10), HBP 15 minutes has better radiomic characteristics in comparison to HBP 5 minutes and HBP 10 minutes. In addition, the case collection and analysis were conducted at the same medical center with the same research methods. Therefore, all feature scoring in our study was based exclusively on HBP 15 min images in previous study. Despite the good performance exhibited by radiomic signatures, it remains a gap compared to clinical radiology models (AUC 0.732 vs. 0.802). We further incorporated radiomics signatures into clinicoradiological model to enhance the predictive power. The subsequence radiomic nomograms displayed modified diagnostic performance, suggesting the higher usefulness of combined approach in MVI prediction in comparison to clinical radiology models. This was consistent with previous study (22, 29), also showing that combined radiomics signatures and clinicoradiological factors should clearly be preferred over clinical risk factors alone in predicting MVI in HCC. For further comparison of the three models, we applied decision curve analysis, which is used for constructing models capable of assessing clinical outcomes and calculating the loss of gain from the assessment model for each individual, largely compensating for the shortcomings of traditional statistical measures (30). In terms of decision curve analysis, the radiomics nomogram proposed in our study is potentially serving to estimate postoperative outcomes in clinic.

In conclusion, the radiomics nomogram successfully presented in our study possesses significant utility in predicting MVI in HCC. It will contribute to providing an important reference for clinicians to protocol the best treatment plan, thereby improving clinical outcomes.

### Limitations

There also exist some limitations in this study. First, this study is a retrospective single-center study, which requires in-depth prospective multicenter validation with a larger cohort. Second, the complex relationship between radiomic signatures and biological behavior fails to be effectively explained.

## Data availability statement

The original contributions presented in the study are included in the article/supplementary material Further inquiries can be directed to the corresponding author.

## Ethics statement

Ethical review and approval was not required for the study on human participants in accordance with the local legislation and institutional requirements. Written informed consent for participation was not required for this study in accordance with the national legislation and the institutional requirements.

## Author contributions

SZ and GW completed the initial manuscript and designed the whole study. CD and RZ collected patients and recorded the needed information. FL and XW collected CT texture features. QS and CD helped collected cases and reviewed the manuscript. XZ and YG provide. All authors contributed to the article and approved the submitted version

## Funding

This study was funded by the clinical medicine +X scientific research project of the Affiliated Hospital of Qingdao University (QDFY+X202101021).

## Conflict of interest

The authors declare that the research was conducted in the absence of any commercial or financial relationships that could be construed as a potential conflict of interest.

## Publisher’s note

All claims expressed in this article are solely those of the authors and do not necessarily represent those of their affiliated organizations, or those of the publisher, the editors and the reviewers. Any product that may be evaluated in this article, or claim that may be made by its manufacturer, is not guaranteed or endorsed by the publisher.
